# Growth and ontogeny of the tapeworm *Schistocephalus solidus* in its copepod first host affects performance in its stickleback second intermediate host

**DOI:** 10.1186/1756-3305-5-90

**Published:** 2012-05-07

**Authors:** Daniel P Benesh, Nina Hafer

**Affiliations:** 1 Department of Evolutionary Ecology, Max Planck Institute for Evolutionary Biology, August-Thienemann-Strasse, 2, 24306, Plön, Germany

**Keywords:** Cercomer, Cestoda, Complex life cycle, Energy allocation, Glycogen, Life history tradeoff, Metamorphosis, Plerocercoid, Procercoid

## Abstract

**Background:**

For parasites with complex life cycles, size at transmission can impact performance in the next host, thereby coupling parasite phenotypes in the two consecutive hosts. However, a handful of studies with parasites, and numerous studies with free-living, complex-life-cycle animals, have found that larval size correlates poorly with fitness under particular conditions, implying that other traits, such as physiological or ontogenetic variation, may predict fitness more reliably. Using the tapeworm *Schistocephalus solidus*, we evaluated how parasite size, age, and ontogeny in the copepod first host interact to determine performance in the stickleback second host.

**Methods:**

We raised infected copepods under two feeding treatments (to manipulate parasite growth), and then exposed fish to worms of two different ages (to manipulate parasite ontogeny). We assessed how growth and ontogeny in copepods affected three measures of fitness in fish: infection probability, growth rate, and energy storage.

**Results:**

Our main, novel finding is that the increase in fitness (infection probability and growth in fish) with larval size and age observed in previous studies on *S. solidus* seems to be largely mediated by ontogenetic variation. Worms that developed rapidly (had a cercomer after 9 days in copepods) were able to infect fish at an earlier age, and they grew to larger sizes with larger energy reserves in fish. Infection probability in fish increased with larval size chiefly in young worms, when size and ontogeny are positively correlated, but not in older worms that had essentially completed their larval development in copepods.

**Conclusions:**

Transmission to sticklebacks as a small, not-yet-fully developed larva has clear costs for *S. solidus*, but it remains unclear what prevents the evolution of faster growth and development in this species.

## Background

Animals with complex life cycles live in distinct habitats as larvae and adults, and switching from one habitat to the next is a critical life history transition. In many taxa, large larvae have higher survival and fecundity as adults (e.g. [1-7]), but, all else equal, it takes longer to grow to a large larval size, increasing the probability of dying before switching. This tradeoff between the benefits of being big and the costs of becoming big is at the heart of many life history models examining optimal switching strategies [8-13]. In these models, fitness is often a function of size and age at the transition. This may turn out to be too simplistic, because a number of studies have found that size and age at metamorphosis can be poor predictors of fitness under some environmental conditions [14-19]. Other factors that are not necessarily correlated with size and age, such as physiological variables, may couple larval and adult success [20-24]. For example, the lifetime mating success of the damselfly *Lestes viridis* is affected not only by size and age at emergence, but also by nutritional and photoperiod treatments whose effects seem mediated by energy stores [25,26].

Many helminth parasites have complex life cycles in which they are trophically-transmitted between consecutive hosts before reproducing. Trait variation in one host often has carryover effects in the next host [27-30], and larval size and age at transmission are prime candidates for predicting such carryover effects [31-36]. Larvae that grow to a large size in the intermediate host generally have higher infection success or fecundity in the next host [37-40]. However, a few studies suggest that the larval size-fitness correlation may depend on environmental factors like the intensity of infection in the intermediate host [41,42] or the size of the intermediate host [43]. Older larvae can also have higher fitness in the next host [44,45]. Older larvae are generally bigger, but potentially also more mature, so it is unclear exactly how this effect arises. Whereas free-living animals transition into the next habitat at a comparable developmental stage, parasites have to wait to be eaten by the next host and may thus be transmitted at an underdeveloped stage with negative consequences for fitness. Elucidating which larval traits reliably affect fitness in the next host is necessary to understand the evolution of life history strategies in complex life cycle parasites [31-33,35,46,47].

Using the tapeworm *Schistocephalus solidus*, we explored the roles of larval size, age, and ontogeny in determining performance in the next host. This tapeworm has a three-host life cycle [48,49]. Adult worms occur in the intestine of fish-eating birds where they mate and release eggs into the environment. Free-swimming larvae hatch from the eggs and are consumed by freshwater copepods, the first host. Tapeworm larvae in copepods, termed procercoids, undergo a period of growth and development before they are infective to three-spined sticklebacks (*Gasterosteus aculeatus*), the second intermediate host. Transmission is trophic, and soon after being consumed by sticklebacks the parasite invades the body cavity [50]. Worms, now dubbed plerocercoids, grow for several weeks in fish before becoming infective to birds [51]. Here, we focused on the transition from copepods to fish. Fitness in fish (infection probability and growth rate) increases with age at transmission [45], and when age is kept constant, big procercoids have higher fitness [43]. However, the correlation between procercoid size and fitness only holds when copepod size is kept rather constant, i.e. being large relative to the host is beneficial, but not necessarily being large in general. Variation in developmental maturity could explain both the effect of age and the effect of relative size. Morphological changes indicative of infectivity occur as establishment probability increases with procercoid age. Moreover, procercoid size and development are positively correlated within copepod stages (relative size correlates with development), but not between them (copepod-stage-induced size variation is not correlated with development) [52,53].

We measured three components of worm fitness in fish (infection probability, growth rate, energy storage) and evaluated how they were related to larval traits (size, age, ontogeny). We exposed fish to worms of two different ages (11 or 17 days in copepods). If size affects fitness mainly through its relationship with ontogeny, then we expected a size-fitness correlation to be steeper in the young group (11 days), because there is more developmental variation at this time. We also kept copepods on either a high or low food diet to 1) induce size variation and to 2) assess whether there are nutritionally-determined carryover effects poorly captured by the other measured larval traits.

## Methods

### Infection protocol and procercoid size measurements

Both the copepods and the tapeworms used in the experiment were raised in the laboratory, but they were originally collected from Lake Skogseidvatnet, Norway (60°13′ N, 5°53′ E). Plerocercoids were dissected from the body cavity of sticklebacks that had been reared and infected in the lab. Worms were bred in size-matched pairs in an in vitro system that was developed by Smyth [54] and later modified by Wedekind [55]. Size-matching facilitates outcrossing [56]. Eggs were collected and stored at 4°C for 1 week, before being incubated at 20° for 3 weeks in the dark. Eggs were exposed to light one day before the copepod exposure to induce hatching.

To produce copepods for the experiment, several tanks (5 L) were set up containing 5–10 egg-bearing female copepods (*Macrocyclops albidus*) (details of the copepod cultures can be found in [57]). After 4 weeks, adult male copepods were collected from these tanks and individually isolated in the wells of a 24-well microtitre plate (~1.5 ml per well). By using only adult male copepods, we eliminated any variation attributable to copepod stage, sex, or growth (adults do not molt further). One day after isolation, each copepod was exposed to a single coracidium. Single-worm infections seem to be the norm for cestode-copepod systems in the field [58-62]. Copepods were maintained at 18°C with an 18:6 L:D cycle, and were fed with either two (low food treatment) or four (high food treatment) *A. salina* nauplii every other day. Copepod survival and parasite growth are reduced in the low food treatment [52], implying these treatments are sufficient to produce variation in the energy available to developing worms.

Copepods are reasonably transparent, permitting worm larvae to be observed in vivo. Nine days post exposure (DPE) infected copepods were placed on a slide, and procercoids were recorded as having or not having a cercomer. The cercomer is a round structure that forms on the posterior end of worms, and although its function is not known, its appearance is correlated with the development of infectivity to fish [63]. Thus, cercomer presence/absence 9 DPE dichotomizes worms into groups of fast or slow developers.

The area of larval worms was measured one day prior to exposing fish (either 10 or 16 DPE). Copepods were placed on a microscope slide and photographed two times. Procercoid area was measured using the freeware Image J 1.38x (Rasband, W.S., NIH, Bethesda, Maryland, USA, http://rsb.info.nih.gov/ij/, 1997–2009) and the two measurements were averaged to give values for individual worms. Area was calculated without the cercomer, because the outline of the cercomer is often difficult to clearly observe in vivo and because cercomer size is tightly correlated with procercoid body size [64]. Thus, calculating worm area with or without the cercomer likely gives very similar results.

### Fish infection and dissection

Lab-bred sticklebacks (7 to 8 months old, mean length = 4.2 cm (± 4.2 SD)) were randomly assigned to be exposed to procercoids that had been in well- or poorly-fed copepods for either 11 or 17 days. At 17 DPE, nearly all procercoids appear morphologically mature, but at 11 DPE there is substantial developmental variation [52,53]. A few days before exposure, fish were individually isolated in small tanks (18 x 13 × 11 cm), and a dorsal spine was clipped to provide DNA for later identification. Each fish was exposed to one infected copepod. Several days after exposure, fish were weighed, measured, and transferred to larger tanks (30 × 22 × 25 cm) at densities of 15 to 17. Three times per week fish were fed ad libitum with frozen chironomids and cladocera. Twenty-five to 28 DPE fish were killed and dissected, and all collected worms were weighed to the nearest 0.1 mg. At this time, worm growth is exponential and apparently unconstrained by fish size [65], so plerocercoid weight reflects variation in growth rates. We took a tail clip for DNA extraction. By taking fish tissue samples both before exposure and after dissection, we were able to identify individual fish, and thus know to which procercoid it was exposed, without maintaining fish in individual tanks. DNA was extracted from spine and tail clips with the Qiagen DNeasy 96 Blood and Tissue Extraction Kit, following the manufacturer’s protocol. Nine microsatellite loci were amplified in two multiplex PCR reactions (conditions given in [66]).

### Glycogen assay

Glycogen is the most important macronutrient for energy storage in tapeworms [63]. We quantified the glycogen content of the young plerocercoids for two reasons: 1) to use as an additional fitness component and 2) to check whether growth rate impacts energy reserves and thus to critically evaluate plerocercoid size as a fitness component. Glycogen content was assayed based on the protocol described by Gómez-Lechón et al. [67]. Plerocercoids were homogenized in a cell mill (Qiagen TissueLyser II, Retsch GmbH). Glycogen standards of known concentration were prepared and run simultaneously (Sigma G0885, concentrations: 900, 700, 500, 300, 200, 100, 50, 0 μg). Samples were diluted to concentrations of ~0.1 to 1.8 μg μl^-1^, and 40 μl per sample were pipetted into the wells of a 96-well microtitre plate. 60 μl of a glucoamylase solution (250 mU/well enzyme [Sigma A1602] in 0.2 M sodium acetate buffer, pH 4.8) were added to each well, and samples were incubated for 2 hr at 40°C with shaking. Plates were then spun at 2500 rpm for 5 min and 10 μl of 0.25 M NaOH were added to stop the enzymatic reaction. To quantify the freed glucose, a Glucose Oxidase/Peroxidase coloring reagent was prepared following the manufacturer’s instructions (Sigma G3660) with 1 mg/ml ABTS (Merck 194430) in 100 mM phosphate buffer, pH 7, included. This coloring reagent was added to each well (200 μl), samples were incubated in the dark for 30 min, and absorbance was recorded at 405 nm with a PowerWave Microplate Spectrophotometer (Bio-Tek Instruments). Samples were run in triplicate. Glycogen values were repeatable within individuals (Intraclass correlation coefficient = 0.995, *P* < 0.001), so they were averaged. Glycogen was expressed as a density (μg per mg plerocercoid fresh weight).

### Data analyses

We separately analyzed three fitness components: infection rate in fish, growth in fish (plerocercoid weight), and the energy reserves of plerocercoids (μg glycogen per mg fresh weight). Infection was analyzed with logistic regression, while general linear models (ANOVA) were used to assess growth and glycogen. We included four predictors in all statistical models: procercoid age at transmission (11 or 17 days, AGE), procercoid size at transmission (PROC), cercomer presence/absence day 9 (fast and slow development, DEVO), and feeding treatment (high and low, FEED). For the analysis of plerocercoid weight and glycogen content, we also included as a factor the number of days worms spent in fish (25, 26, 27, or 28). All main effects were tested as well as the following potentially interesting interactions AGE x DEVO (is developmental variation measured 9 DPE particularly important at a young age?), AGE x PROC (does size only matter when there is developmental variation early on?), and AGE x FEED (does time spent in the feeding treatments matter?). Preliminary analyses and previous studies [43] indicated that characteristics of the fish host, such as its size, sex, and condition (hepatosomatic index), did not influence the measured fitness components, so they were not considered.

Statistical analyses were conducted with SPSS 18.0 (SPSS Inc., Chicago, Ill.) and R 2.14.1 (R Development Core Team, Vienna, Austria). The dataset is available as Additional file 1. We also re-visited some of the results of Benesh et al. [43]. They studied how *S. solidus* procercoid size 14 DPE affects infection probability and growth in fish. This is between our two age groups (11 and 17 DPE), so we present their results for comparative purposes. Note that due to the different experimental conditions we did not jointly analyze data from Benesh et al. and the current experiment. Cercomer presence/absence 9 DPE had been recorded in the previous study, but its importance was not evaluated. Plerocercoid size measurements in the two studies are not easily comparable (fully-developed worms vs the young, growing plerocercoids studied here), so we only show infection rate results. The results from their experiments using large copepods are presented, as that is most comparable to this study.

## Results

### Determinants of infection

**Figure 1:**
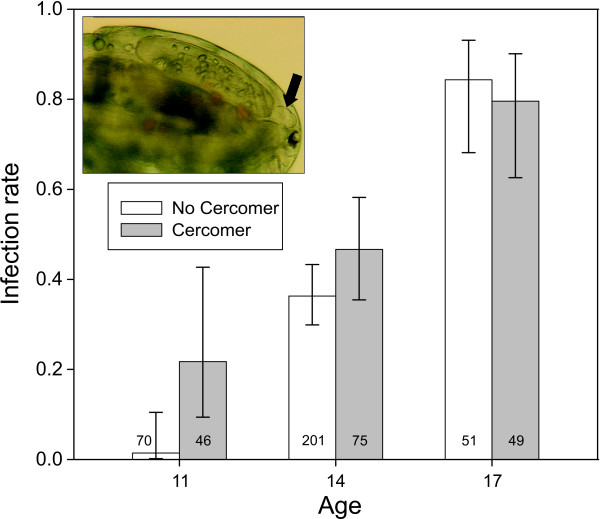
**Infection rate of procercoids after 11, 14, or 17 days in copepods.** Procercoids were recorded as having or not having a cercomer on day 9. Inset photograph shows a procercoid with a well-developed cercomer in vivo (arrow). Error bars represent the 95% CI and numbers within columns are sample sizes. Data from day 14 were from ref [43].

**Figure 2:**
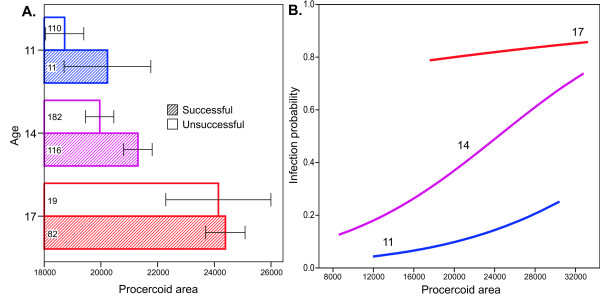
**(A) The mean size of procercoids (um**^**2**^**) that did (hatched bars) and did not (open bars) successfully infect sticklebacks after 11, 14, or 17 days in copepods.** Error bars represent the 95% CI and numbers within columns are sample sizes. Data from day 14 were from ref [43]. (B) The relationship between infection probability and procercoid area predicted by logistic regressions performed separately for each of the three age groups. Only the regression at day 14 was statistically significant.

**Figure 3:**
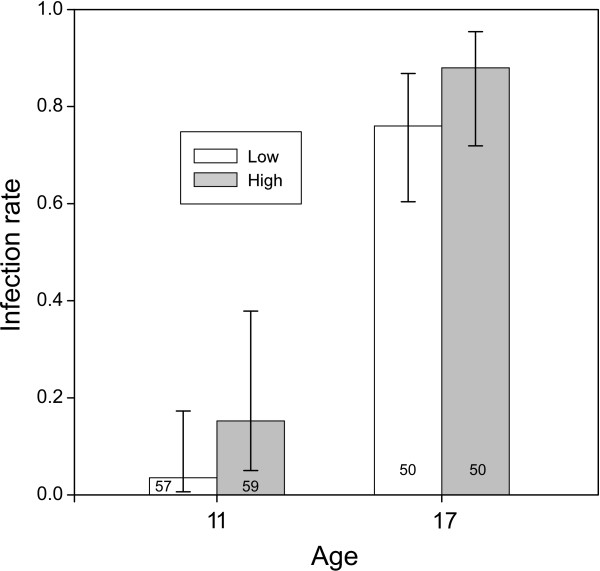
**Infection rate of procercoids in the high and low feeding treatments after 11 and 17 days in copepods.** Error bars represent the 95% CI and numbers within columns are sample sizes.

At 11 DPE, only 9.5% (11/116) of procercoids successfully infected fish, whereas 82% (82/100) were successful at 17 DPE. Given the low variation in infection success within the two age groups, there was relatively low power to detect interactions between AGE and the other predictors, so non-significant effects need to be interpreted cautiously. There was, however, a significant AGE x DEVO interaction (Wald χ_1_^2^ = 5.92, *P* = 0.015). Fast developers had a higher infection probability 11 DPE, but not 17 DPE, and the results of Benesh et al. suggest an intermediate effect 14 DPE (Figure 1). Surprisingly, neither PROC nor its interaction with AGE was significant (Wald χ_1_^2^ = 0.054, P = 0.82 and Wald χ_1_^2^ = 0.026, *P* = 0.87, respectively), even though bigger worms seemed more successful at day 11 (and day 14) (Figure 2). Similarly, FEED seems important when considered in isolation, with procercoids from the low food treatment having lower infection rates (Figure 3), but the effect was not significant in the full model (Wald χ_1_^2^ = 2.23, *P* = 0.135). The PROC and FEED main effects remained non-significant when their interactions with AGE were removed from the model (*P* = 0.84 and *P* = 0.09, respectively). The absence of significant PROC or FEED effects could reflect collinearity, i.e. the variation in infection attributable to these variables is better captured by AGE or DEVO.

### Determinants of plerocercoid size

**Figure 4:**
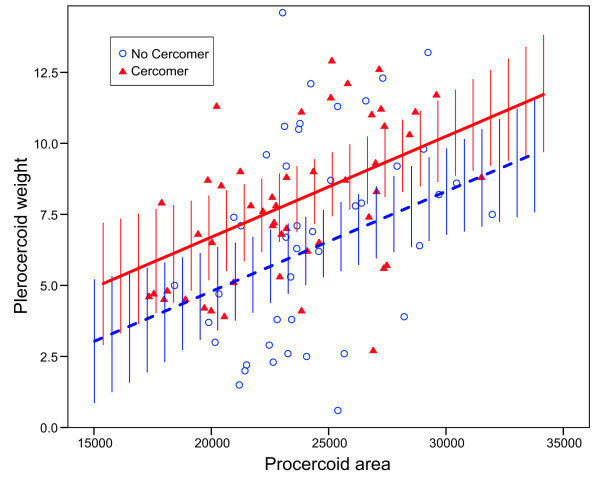
**The relationship between procercoid area (um**^**2**^**) in copepods and plerocercoid weight (mg) in fish.** The best fit regression, estimated by the general linear model with just main-effects, was plotted separately for procercoids that did (filled triangles, solid line) and did not (open circles, dashed line) have a cercomer after 9 days in copepods. Bars around regression lines are the 95% CI.

No terms significantly affected plerocercoid weight in the full model (all *P* > 0.05), with the exception of days in fish (*P* = 0.038). However, this model was significantly better than an intercept-only model (R^2^ = 0.306, F_8, 84_ = 3.62, *P* < 0.001), suggesting additional variables had explanatory value. Because 88% (82/93) of the worms recovered from fish were from 17 DPE, the interactions between AGE and the other predictors were estimated with large standard errors. Removal of the interaction terms one-by-one did not significantly reduce explanatory power and result in a worse model (all F_1, 84_ < 0.692, all *P* > 0.41, R^2^ dropped from 0.306 to 0.294 with all interactions removed). A model with only the five main effects indicated that plerocercoid size increased with days in fish (F_3, 85_ = 2.89, P = 0.04), that it increased with procercoid size (F_1, 85_ = 12.92, *P* = 0.001), and that worms with a cercomer 9 DPE grew to be larger plerocercoids (F_1, 85_ = 9.68, *P* = 0.003) (Figure 4). AGE and FEED were not significant (F_1, 85_ = 0.18, *P* = 0.67 and F_1, 85_ = 0.14, *P* = 0.71, respectively).

### Determinants of energy content

**Figure 5:**
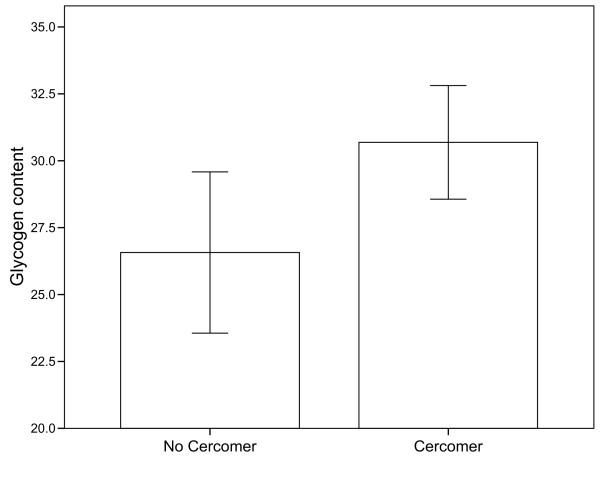
**Mean glycogen content (ug mg**^**-1**^**fresh weight) of plerocercoids from fish that had developed fast (cercomer after 9 days in copepods) or slow (no cercomer on day 9) in copepods.**

None of the two-way interactions had a significant effect on glycogen content (all F_1, 80_ < 2.84, all *P* > 0.096), and their removal did not significantly decrease explanatory power (R^2^ dropped from 0.191 to 0.158, F_3, 80_ = 1.09, P = 0.36). A main-effects-only model indicated that plerocercoids had higher glycogen content if they had a cercomer 9 DPE (F_1, 83_ = 9.61, *P* = 0.003) (Figure 5). There was also a non-significant trend for worms from the high feeding treatment to have more glycogen (F_1, 83_ = 3.17, *P* = 0.079), but all other effects were not significant (all F < 2.12, all *P* > 0.15).

**Figure 6:**
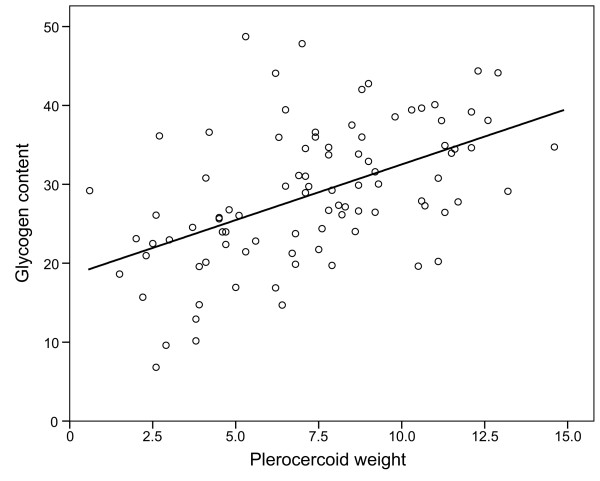
**Scatterplot of plerocercoid weight (mg) versus glycogen content (ug mg**^**-1**^**fresh weight) with the least squares regression line.**

It should also be noted that plerocercoid size and glycogen content were positively correlated (R^2^ = 0.259, F_1, 90_ = 31.2, *P* < 0.001) (Figure 6).

## Discussion

How long [45] and how fast [43]*S. solidus* grow in copepods is known to influence infection and growth in sticklebacks. Our results complement those studies and suggest that larval ontogeny is very important for the coupling of performance in the two hosts. Procercoids that develop faster (have a cercomer 9 DPE) are able to infect fish sooner, and they tend to grow to larger sizes with larger energy reserves in fish. Moreover, the previously documented association between procercoid size and fitness seems partially attributable to ontogeny. Fast-growing procercoids tended to have higher infection rates at 14 DPE, and perhaps 11 DPE. Ontogeny is positively correlated with procercoid size at this time, at least when environmental conditions (copepods stage) are constant [52,53], suggesting that developmental variation may underlie the increase in infection probability with procercoid size. At 17 DPE worms are essentially fully developed, and at this time point there was no influence of procercoid size or ontogeny on infection probability. On the other hand, worms that were bigger when entering fish were also bigger when recovered, suggesting that procercoid size may influence fitness independently of ontogeny.

Growth and development are interwoven processes, so their individual contributions to fitness are difficult to completely disentangle. It is nonetheless clear that worms that grow and develop rapidly have the highest fitness under experimental settings. For example, let us compare a fast-growing, fast-developing worm (cercomer 9 DPE and one SD larger than the sample mean) with a slow-growing, slow-developing worm (no cercomer 9 DPE and one SD smaller than the mean). The fast-growing worm is predicted to have up to 20% higher infection probability (at 11 DPE), and to be ~85% bigger with ~25% higher glycogen content after 3.5 weeks in sticklebacks. Hammerschmidt et al. [45] suggested that the optimal switching time for *S. solidus* balances increasing establishment probability in fish and decreasing survival probability in copepods. This is similar to the size-age tradeoffs thought to shape switching times in free-living animals with complex life cycles. Just as the tradeoff between size and age depends on growth rate [68], the tradeoff between establishment probability and mortality is mediated by developmental rate; worms that rapidly develop may switch earlier to fish, avoiding age-related mortality in copepods. Thus, the advantages to rapid growth and development appear pronounced: earlier infectivity and the resulting avoidance of mortality in copepods as well as faster and more efficient growth in fish.

Although there seems to be selection for rapid growth and development in copepods, long-term phenotypic change is unlikely. Parasite species from divergent taxa with similar life cycles (e.g. transmission from a copepod to a fish) tend to exhibit characteristic rates of larval growth and development, strongly suggesting life history strategies converge to universal adaptive peaks for a given type of life cycle [69]. Thus, there are presumably important tradeoffs that make extremely rapid growth or development suboptimal. Several hypotheses exist: 1) rapid growth and ontogeny requires over-consumption of host nutrients reducing host survival (i.e. the virulence tradeoff [31,70]), 2) acquiring the resources for rapid growth and ontogeny requires host specialization and reduced generality [71], 3) rapid growth or ontogeny is less efficient, resulting in higher susceptibility to environmental stressors, such as starvation [26,72,73], 4) maturation, and the cell differentiation it entails, reduces growth potential [74]. Benesh [75] argued that there is relatively little evidence for hypothesis one for the larval stages of trophically-transmitted helminths, including *S. solidus* in copepods [55,57,76]. Hypothesis two cannot be discounted, because host specificity seems important for the larval life history of some tapeworms [77], although *S. solidus* is a generalist in copepods [78]. Hypotheses three and four are allocation tradeoffs (somatic growth vs energy storage, maturation vs growth potential). Such tradeoffs can be masked by variation in resource acquisition [79,80]. For instance, in opposition to hypothesis four, fast-growing *S. solidus* procercoids also develop quicker [53], perhaps because they have more resources available to them. Certainly there is more work to do to identify the tradeoffs shaping larval life history strategies in parasites.

The feeding treatment had only moderate, non-significant effects on infection rate and glycogen content, and no effect on plerocercoid size. A possible explanation for this is that, given their stronger effects, procercoid size and cercomer presence/absence explain the variation in fitness induced by the feeding treatment. In any case, carryover effects attributable to unmeasured condition variables do not appear to be pronounced. Some of the covariance between larval traits (growth and ontogeny) and the fitness components was surely induced by the feeding treatment and thus environmentally-determined. Because genetic variation is a prerequisite for trait evolution, it will be interesting to see if there is genetic covariance between larval traits and fitness, i.e. do parasite genotypes that rapidly grow and develop also have higher infection probability?

Glycogen makes up approximately 16% of the weight of fully-developed plerocercoids taken from fish (>100 mg) [81]. In the young plerocercoids studied here (~7 mg on average), glycogen constituted 2.9% of worm wet weight, and in medium-sized plerocercoids (~75 mg) it is about 10% (Benesh and Kalbe, unpublished data). Thus, worms appear to steadily increase their glycogen reserves as they grow in fish. We observed that the fastest-growing worms in fish had the highest glycogen content, suggesting rapid growth is not inefficient and contradicting hypothesis three above. This may be another case in which variation in resource acquisition masks an allocation tradeoff, i.e. worms in good condition can invest in both somatic growth and energy storage.

## Conclusions

Transmission up the food web into bigger, ‘better’ hosts does not imply a new start for parasites. Analogous to free-living organisms with complex life cycles, phenotypic variation in the intermediate host can have carryover effects in the next host, though additional studies are needed to generalize this. For *S. solidus* procercoids, transmission to sticklebacks as a small, not-yet-fully developed larva has clear costs in terms of lower infection probability and stunted, inefficient growth. Given the seemingly strong selection for rapid growth and development in copepods, more work is needed to identify what prevents change in the ontogenetic schedule of *S. solidus* (ecological tradeoffs? genetic constraints? developmental thresholds?).

## Competing interests

The authors declare that they have no competing interests.

## Authors’ contributions

DPB conceived the study. DPB and NH performed the experiment, collected the data, and outlined the manuscript. DPB wrote the manuscript. Both authors read and approved the final manuscript.

## Supplementary Material

Additional file 1 **Description of the data file for the paper "Growth and ontogeny of the tapeworm Schistocephalus solidus in its copepod first host affects performance in its stickleback second intermediate host".** The columns in the data file "Benesh_Hafer_datafile.csv" are listed below with descriptions of their contents.AGE - Age of worms when given to fish (either 11 or 17 days after infecting copepods).FEED - Infected copepods were fed either a high (H) or low (L) food diet.CERC - Whether worms did (1) or did not (0) have a cercomer after 9 days in copepods.PROC - The size of procercoids (um2) measured in vivo one day before exposure to fish.I.FTL - Total length (mm) of fish at exposure.I.FSL - Standard length (mm) of fish at exposure.I.FW - Weight (g) of fish at exposure.DAYS - Days between exposure and dissection of fish.F.FTL - Total length (mm) of fish at dissection.F.FSL - Standard length (mm) of fish at dissection.F.FW - Weight (g) of fish at dissection.Corr.F.FW - Fish weight when the weight of the plerocercoid is removed.>LW - Weight (mg) of the liver.HSI - Hepatosomatic index (LW/Corr.F.FW).INF - Whether the worm successfully infected a fish (1) or not (0).PLER - Weight (mg) of plerocercoids recovered from fish.SEX - Sex of fish (0 = male, 1 = female).GLYC - Glycogen content of recovered plerocercoids (ug glycogen per mg fresh weight).REMARK - Two samples were lost during glycogen extraction, as noted in this column. Click here for file
